# Vaccine and Immunotherapeutic Approaches for the Prevention of Cryptococcosis: Lessons Learned from Animal Models

**DOI:** 10.3389/fmicb.2012.00291

**Published:** 2012-08-28

**Authors:** Camaron R. Hole, Floyd L. Wormley

**Affiliations:** ^1^Department of Biology, The University of Texas at San AntonioSan Antonio, TX, USA; ^2^The South Texas Center for Emerging Infectious Diseases, The University of Texas at San AntonioSan Antonio, TX, USA

**Keywords:** *Cryptococcus neoformans*, cryptococcosis, *Cryptococcus*, fungal pathogenesis, host-fungal interactions, fungal vaccines

## Abstract

*Cryptococcus neoformans* and *C. gattii*, the predominant etiological agents of cryptococcosis, can cause life-threatening infections of the central nervous system in immunocompromised and immunocompetent individuals. Cryptococcal meningoencephalitis is the most common disseminated fungal infection in AIDS patients, and *C. neoformans* remains the third most common invasive fungal infection among organ transplant recipients. Current anti-fungal drug therapies are oftentimes rendered ineffective due to drug toxicity, the emergence of drug resistant organisms, and/or the inability of the host’s immune defenses to assist in eradication of the yeast. Therefore, there remains an urgent need for the development of immune-based therapies and/or vaccines to combat cryptococcosis. Studies in animal models have demonstrated the efficacy of various vaccination strategies and immune therapies to induce protection against cryptococcosis. This review will summarize the lessons learned from animal models supporting the feasibility of developing immunotherapeutics and vaccines to prevent cryptococcosis.

## Introduction

*Cryptococcus neoformans and C. gattii*, the etiological agents of cryptococcosis, are encapsulated fungal pathogens that cause fungal pneumonia and life-threatening infections of the central nervous system (CNS) (Mitchell and Perfect, 1995). Cryptococcal meningoencephalitis remains the most common disseminated fungal infection in AIDS patients (Powderly, 1993). Global estimates suggest that one million cases of cryptococcal meningitis due predominantly to *C. neoformans* occur each year resulting in approximately 620,000 deaths (Park et al., 2009). *C. gattii* primarily causes life-threatening fungal meningitis and infections of the lung and skin in otherwise healthy individuals (Jarvis and Harrison, 2008). The geographical distribution of *C. gattii* was believed to be limited to tropical and subtropical climates such as Australia, New Zealand, and Southeast Asia (Kwon-Chung and Bennett, 1984). However, *C. gattii* infections began to occur within animal and human populations on Vancouver Island, British Columbia, Canada, a temperate climate. Subsequently, *C. gattii* disseminated onto the mainland of British Columbia and proceeded throughout the Pacific Northwest of the United States (Datta et al., 2009a,b; Centers for Disease Control and Prevention, 2010; Galanis and Macdougall, 2010).

The administration of highly active antiretroviral therapy (HAART) has resulted in a decrease in the number of cases of AIDS-related cryptococcosis in developed countries, but in developing countries where HAART is not readily available, *Cryptococcus* is still a major problem (Dromer et al., 2004). Those who are successfully treated for AIDS-associated cryptococcal meningitis oftentimes require life-long anti-fungal therapy due to a high relapse rate (Bozzette et al., 1991; Vibhagool et al., 2003). *C. neoformans* also remains the third most common invasive fungal infection among organ transplant recipients (Husain et al., 2001; Singh et al., 2005; Pappas et al., 2010). Studies have shown that 2.8% of organ transplant recipients can develop cryptococcal infections resulting in an overall estimated death rate of 42% (Husain et al., 2001). Also, cryptococcosis accounted for 8% of the invasive fungal diseases in solid organ transplant recipients in the Transplant Associated Infection Surveillance Network database from March 2001 to March 2006 (Pappas et al., 2010). The acute mortality rate is between 10 and 25% in medically advanced countries (Powderly, 1993), and at least one third of patients with cryptococcal meningitis who receive appropriate therapy will undergo mycologic and/or clinical failure (Van Der Horst et al., 1997; Saag et al., 2000). Thus, there remains an urgent need for therapies and/or vaccines to combat cryptococcosis.

The current clinical picture of cryptococcosis suggests that immunosuppressed patients or individuals predicted to be at an exceptionally high risk for developing cryptococcosis are likely targets for vaccination to prevent the development of cryptococcal disease. Additional target populations for vaccination as a prophylactic measure include immune competent persons in high exposure areas as highlighted by the recent *C. gattii* outbreak in Vancouver Island, British Columbia, Canada, or those at high risk for exposure to HIV. Animal models of cryptococcosis have been important tools for elucidating the mechanisms of protection and for testing the efficacy of various antibody or antigen-based vaccine candidates. This review will focus on the lessons learned from animal models of cryptococcal disease that support efforts toward developing immunological therapies and/or vaccines against cryptococcosis.

## Host Response to *Cryptococcus*

The consensus is that cell-mediated immunity (CMI) primarily by Th1-type CD4^+^ T cells directs the protective host immune response against *C. neoformans* (Huffnagle et al., 1991; Casadevall, 1995; Zhang et al., 2009), whereas a Th2-type immune response is considered detrimental to the host (Arora et al., 2005; Jain et al., 2009). A Th1-type response is characterized by the production of interleukin (IL)-2, IL-12, interferon-gamma (IFN-γ), and tumor necrosis factor-alpha (TNF-α) (Cherwinski et al., 1987). These cytokines induce granuloma formation in the lung to wall off the yeast and are responsible for initiating delayed-type hypersensitivity (DTH) reactions that lead to enhanced uptake, killing by neutrophils and dendritic cells, and the induction of classically activated macrophages (Cher and Mosmann, 1987; Zhang et al., 2009; Arora et al., 2011). A Th2-type response is characterized by the production of IL-4, IL-5, IL-9, IL-10, and IL-13 (Cherwinski et al., 1987), and is associated with eosinophilic inflammation and dissemination of cryptococci to the CNS (Huffnagle et al., 1994; Olszewski et al., 2001; Muller et al., 2007). A Th17-type response consisting of IL-17, IL-21, and IL-22 (reviewed in Onishi and Gaffen, 2010) has been suggested to be important in protection against *C. neoformans*, however, it seems to play a secondary role to the Th1-type response (Kleinschek et al., 2010). When a Th17-type response is blocked, there is no effect on survival of mice with experimental pulmonary *C. neoformans* infection (Wozniak et al., 2011a).

The predominant studies evaluating host immune responses to cryptococcal infections have been performed using *C. neoformans* as the model organism. However, several studies now suggest that the mechanism for mediating protection against *C. neoformans* infections differs from that which induces protection against *C. gattii*. Experimental studies suggest that *C. gattii* may thrive in immune competent hosts by suppressing host immune responses (Dong and Murphy, 1995; Wright et al., 2002; Cheng et al., 2009; Kronstad et al., 2011). *C. gattii* strain R265, the predominant isolate of the Vancouver Island outbreak, has been shown to be more suppressive in mice compared to those infected with *C. neoformans. C. gattii* infected mice had decreased neutrophil recruitment and decreased pro-inflammatory cytokine production (Dong and Murphy, 1995; Wright et al., 2002; Cheng et al., 2009).

While the patient population that historically develops cryptococcal infections includes individuals with defects in CMI, patients with defects in antibody-mediated immunity (AMI) such as hypogammaglobulinemia and hyper-IgM syndrome also have an increased probability of developing cryptococcosis (Gupta et al., 1987; Iseki et al., 1994; Tabone et al., 1994; Casadevall, 1995; Antachopoulos et al., 2007). The role of AMI in cryptococcal infections is complicated in that antibodies can be protective, non-protective, or can exacerbate the infection (Casadevall, 1995; Mukherjee et al., 1995a,b; Yuan et al., 1998). Antibody protection against cryptococcosis is dependent on the antibody isotype, concentration, and specificity (reviewed in Casadevall, 1995). Comparisons of variable-region-identical mAbs to the glucuronoxylomannan (GXM) component of the *C. neoformans* polysaccharide capsule of the murine IgG_1_, IgG_2a_, IgG_2b_, and IgG_3_ isotypes have consistently shown that all IgG isotypes, except IgG_3_ are protective against *C. neoformans* infection in mice (Yuan et al., 1995, 1998; Nussbaum et al., 1996). Resistance to disease may depend upon the proportion of protective anti-cryptococcal antibodies produced during infection. However, the efficacy of anti-cryptococcal antibodies of a protective isotype can be lost if induced or passively administered in excess creating a prozone-like effect (Taborda and Casadevall, 2001; Taborda et al., 2003). Other major determinants of the effectiveness of antibody isotype against *C. neoformans* infection include mouse genetics (Rivera and Casadevall, 2005; Zaragoza et al., 2007), T cell function (Yuan et al., 1997), and the presence of Th1- and Th2-related cytokines (Beenhouwer et al., 2001). Despite what we know from animal studies concerning protective antibody isotypes, the efficacy of various immunoglobulin isotypes against *C. neoformans* may vary between humans and mice (Beenhouwer et al., 2007) necessitating some caution in trying to translate findings in animal models to the clinic. Nevertheless, anti-cryptococcal antibodies can act as opsonins, participate in antibody-dependent cellular cytotoxicity, augment Th1-type polarization, and help to eliminate immunosuppressive polysaccharide antigen from serum and tissues. They also inhibit cryptococcal biofilm formation, alter lipid metabolism resulting in increased sensitivity to anti-fungal drugs, have direct anti-fungal activity, and modulate the immune response to prevent host damage (Martinez and Casadevall, 2005; Casadevall and Pirofski, 2007; Mcclelland et al., 2010; Brena et al., 2011). The role of antibodies in the host defense against fungal infection remains controversial; however, some recent studies continue to show a protective role of antibodies against cryptococcosis. For example, naturally occurring IgM (nIgM) is essential for the prevention of cryptococcosis in mice (Subramaniam et al., 2010). Cryptococcal disease is more severe in mice lacking serum IgM and in sIgM knockout mice. Experimental pulmonary *C. neoformans* infection of sIgM knockout mice results in increased mortality and higher blood and brain fungal burden compared to that observed in wild-type infected mice. While it is generally agreed that the protective host immune response to *C. neoformans* is dependent on inducing protective CMI, AMI certainly contributes in the protection against cryptococcosis.

## Cryptococcal Protein Targets

Significant efforts have been made towards identification of cryptococcal antigens that induce *Cryptococcus*-specific Th1-type immune responses. This effort is based on the predominant role for CMI responses to mediate protection against *C. neoformans* infections. Protein antigens can induce T-dependent immune responses and thus be highly immunogenic. Additionally, proteins are biochemically defined, thus allowing for the production of large quantities of recombinant proteins. There is also no fear of protein “reversion” that could possibly occur using an attenuated strain to induce immunity. Immunization of mice with *C. neoformans* culture filtrate antigen (CneF), which contains secreted proteins as well as cell wall-associated proteins, resulted in the stimulation of DTH responses (Murphy et al., 1988). Fractionation of CneF revealed its mannoprotein (MP) fraction as the primary antigenic component responsible for DTH and T-dependent immune responses in mice (Murphy et al., 1988; Levitz and Specht, 2006). Mice given two immunizations of purified cryptococcal MP (MP98) experienced prolonged survival and had reduced CFUs in the organs when compared to the control mice receiving adjuvant alone (Mansour et al., 2002). Cryptococcal MP induced Th1-type responses in immunized mice upon infection with *C. neoformans* (Mansour et al., 2002). Four protective MP, one being a chitin deacetylase (MP98) (Levitz et al., 2001), one of unknown function (MP88) (Huang et al., 2002), and an additional two, MP84 and MP115 (Biondo et al., 2005) have been found.

In addition to the MP found in the cell wall, other potential targets include the heat shock protein (hsp) family (Kakeya et al., 1997; Nooney et al., 2005), fungal glucosylceramide (Rodrigues et al., 2007), and melanin (Rosas et al., 2001). MAbs to melanin have been shown to prolong the survival of and reduce fungal burden in lethally infected mice and reduce the growth rate of the fungus *in vitro* (Rosas et al., 2001). In the hsp family, hsp70 has been found to be highly immunogenic (Kakeya et al., 1997). Mycograb, a recombinant antibody targeting an epitope within the hsp90 of *Candida albicans* that is conserved in *Cryptococcus*, leads to enhanced fungicidal activity when combined with amphotericin B treatment of infected mice (Nooney et al., 2005). Targeting antigens common to multiple fungal species such as hsps or melanin may serve to extend protection to multiple disparate fungal pathogens.

## Cytokine Immunotherapies

Adaptive immunity against cryptococcosis is orchestrated via cytokine/chemokine production by Th1-type CD4^+^cells. Consequently, the historic objective of most immune-based therapies has been to skew the non-protective immune responses toward a protective Th1-type immune response. For example, IL-2 given along with a CD40 agonist antibody significantly prolonged the survival of *C. neoformans-*systemically infected mice (Zhou et al., 2006, 2007). In addition, mice depleted of CD4^+^ T cells were protected when stimulated with CD40 and IL-2 compared to depleted mice without these stimulations. This model also showed significant reduction of fungal burden in the organs and increased levels of IFN-γ and TNF-α (Zhou et al., 2006, 2007). Furthermore, mice treated with recombinant TNF-α experienced significantly prolonged survival times compared to non-treated control mice (Kawakami et al., 1996a). Additionally, treatment of mice with a TNF-α-expressing adenoviral vector after experimental challenge of *C. neoformans* resulted in a significant decrease in fungal burden, an increase in IFN-γ levels, and an increase in infiltration of macrophages and neutrophils (Milam et al., 2007). Treatment with IL-12 also led to significant increases in survival of mice with experimental cryptococcosis, but the timing of the treatment is important. Treating mice with IL-12 for 7 days post infection resulted in 90% survival, but delaying IL-12 treatment until day 7 post infection negated the effect of the IL-12, and the mice had similar survival rates compared to the untreated mice (Kawakami et al., 1996c). Administering IL-23, a cytokine necessary for the proliferation and stabilization of the Th17-type cells (reviewed in Onishi and Gaffen, 2010), significantly extended the survival of intraperitoneally infected mice (Kleinschek et al., 2010). However, when compared to their positive control group that received IL-12 and showed 100% survival at day 100, it was concluded that IL-23 may play only a limited role in the protection against *C. neoformans* (Kleinschek et al., 2010). Treating mice with IL-2 and anti-CD40, TNF-α, or IL-12 led to an increase in IFN-γ levels (Kawakami et al., 1996c; Zhou et al., 2006; Milam et al., 2007). Giving mice daily injections of IFN-γ prolonged their survival and reduced the fungal burden in the lungs and the brain (Kawakami et al., 1996b).

Studies in humans indicated that the cytokine profile at the site of infection determines the rate of clearance. Patients with higher IFN-γ at the site of infection had a faster rate of clearance compared to those with lower levels (Siddiqui et al., 2005), suggesting that the microenvironment at the site infection is more important than the macroenvironment. Considering this study, a highly virulent strain of *C. neoformans*, H99, was modified to produce murine IFN-γ. Mice given an experimental pulmonary infection with the IFN-γ producing *C. neoformans* strain, designated H99γ, were capable of completely resolving the infection and showed 100% protection upon re-challenge with wild-type cryptococci (Wormley et al., 2007). This protection was shown to be T cell dependent and B cell independent (Wozniak et al., 2009). T cell knock out (KO) mice succumbed to an experimental pulmonary infection with H99γ whereas B cell KO mice survived and showed 100% protection upon challenge with the wild-type *C. neoformans* strain H99 (Wozniak et al., 2009). Mice immunized with the H99γ strain and then depleted of either CD4^+^ or CD8^+^ T cells prior to and during subsequent challenge with wild-type *C. neoformans*, remained 100% protected (Wozniak et al., 2009). Remarkably, when the same experiment was performed with depletion of both CD4^+^ and CD8^+^ T cells during the challenge with wild-type cryptococci, there was still 80% survival (Wozniak et al., 2011b). These results suggest that if immune competent individuals are immunized against *C. neoformans* and then become T cell deficient they may still be protected. This system also produced high amounts of IL-17A, but when IL-17A deficient or IL-17A receptor KO mice were immunized with the H99γ strain there was no difference in protection compared to the wild-type mice (Hardison et al., 2010; Wozniak et al., 2011a). Thus, IL-17A may contribute to, but ultimately be dispensable for, protection against *C. neoformans* infection.

Cytokine treatment has also shown promising results when administered along with conventional anti-fungal drugs. In a systemic infection model, treating mice with IL-12 along with fluconazole significantly lowered the fungal burden in the brain compared to mice treated with fluconazole alone (Clemons et al., 1994). Administering IFN-γ along with amphotericin B during a lethal systemic infection model significantly lowered the fungal burden in the brain compared to amphotericin alone (Lutz et al., 2000). In a sub-lethal systemic infection model amphotericin B with IFN-γ led to complete clearance of the infection (Lutz et al., 2000). Fluconazole and IFN-γ significantly lowered the fungal burden in both the lethal and sub-lethal systemic infection models but failed to completely resolve the infection in any of the models (Lutz et al., 2000). Cytokine treatment may be necessary considering the immune suppressed status of the typical cryptococcosis patient. However, the possible induction of *C. neoformans*-related immune reconstitution inflammatory syndrome (IRIS) which is the induction of over-exuberant pro-inflammatory responses following the resolution of immune suppression necessitates caution when proceeding with such approaches.

## Antibody-Mediated Immunotherapies and Antibody Vaccines

One of the main virulence factors of *Cryptococcus* is the polysaccharide capsule that is comprised primarily of the polysaccharides GXM and galactoxylomannan (GalXM) and to a much lesser extent, <1%, MP (Zaragoza et al., 2009). Cryptococcal polysaccharides such as GXM have profound suppressive effects on immune responses and elicit little to no antibody responses making it an unlikely choice as an antigen (reviewed in Zaragoza et al., 2009). GXM can bleb off the fungus and direct immune responses away from infection, while GXM and GalXM both can non-specifically diminish lymphocyte proliferation and induce apoptosis (Yauch et al., 2006; Cutler et al., 2007; Chow and Casadevall, 2011; Vecchiarelli et al., 2011). However, an entirely plausible strategy to induce protection against cryptococcosis may be to develop antibodies against specific capsular components. Mouse models have been utilized to investigate the efficacy of cryptococcal conjugate vaccines in which GXM or GalXM has been conjugated to protein carriers (Casadevall et al., 1992, 1998; Devi, 1996; Chow and Casadevall, 2011). GXM conjugated to the tetanus toxoid resulted in anti-GXM protective antibody responses and antibodies specific for GXM which help protect against cryptococcosis (Casadevall et al., 1992, 1998; Devi, 1996). Two GalXM conjugates where tested and found to be useful in generating high titers of anti-GalXM antibodies in the serum, but the responses were not protective (Chow and Casadevall, 2011). Casadevall developed an antibody to the capsule, 18B7, which was found to bind to all serotypes, enhance phagocytosis, and help with the clearance of the blebs of polysaccharide released by the fungus (Casadevall, 1995). Recently, MAb 18B7 has been conjugated to the therapeutic radioisotopes ^188^Rhenium or ^213^Bismuth (Dadachova et al., 2003; Bryan et al., 2010) and studied as a potential radioimmunotherapy (RIT) against *C. neoformans*. Administration of radiolabeled MAb 18B7 to lethally infected mice resulted in prolonged survival, reduced organ fungal burden, and was a more effective therapy compared to mice treated with amphotericin B alone.

A potential strategy to circumvent the immunosuppressant effects of GXM and GalXM may be to focus on specific epitopes within the polysaccharides that elicit protective antibody responses. An approach using small peptides that mimic defined GXM or GalXM epitopes may elicit protective antibody responses where using total polysaccharides has immunosuppressive effects. Peptides which are able to induce antibodies that are capable of binding to the native antigen when administered as immunogens are termed mimotopes. A peptide mimetic (P13) of GXM was developed that is recognized by human anti-GXM antibodies (Zhang et al., 1997). Vaccination with P13-protein conjugates in mice elicited an antibody response to GXM (Zhang et al., 1997). Additionally, mice immunized with the conjugates experienced prolonged survival after an otherwise lethal *C. neoformans* challenge compared to controls (Fleuridor et al., 2001) or following establishment of a chronic infection (Datta et al., 2008). Immunization with P13-protein conjugates also prolonged survival in mice transgenic for human immunoglobulin loci (Fleuridor et al., 2001) and passive immunization with serum from P13-conjugate vaccinated mice to naïve mice conferred partial protection to a lethal cryptococcal challenge (Maitta et al., 2004; Datta et al., 2008). Nonetheless, the therapeutic efficacy of conjugate vaccines containing a peptide mimotope of GXM appears to depend on the genetic background of the host and the carrier protein employed (Datta et al., 2008). Conjugation of P13 to keyhole limpet marine mollusk (KLH) elicited an anti-GXM response to non-protective epitopes possibly due to the generation of non-protective antibodies against an epitope within the carrier protein that is cross-reactive to GXM (May et al., 2003).

Development of antibodies targeting essential components within the fungal cell wall may be an additional method of antibody-mediated protection against cryptococcosis. Treatment with an anti-β-glucan antibody, MAb 2G8, which targets β(1,3) glucans of the brown algae, *Laminaria digitata*, was shown to inhibit capsule formation and growth of *C. neoformans in vitro* and mediate a significant reduction in fungal burden in mice given an experimental systemic cryptococcal infection (Rachini et al., 2007). Also, passive immunization with MAbs against melanin or glucosylceramide prolonged the survival of mice given a lethal infection with *C. neoformans* (Rosas et al., 2001; Rodrigues et al., 2007). Again, targeting fungal antigens that are common among multiple fungal pathogens is an appealing strategy toward generating cross-protection using one immune-therapy and/or vaccine.

## Conclusion

The need for novel treatments to combat cryptococcosis is expected to rise. This is due to an increasing population of immune compromised patients, a high relapse rate, and increased percentage of patients predicted to experience mycologic and/or clinical failure. Animal models of cryptococcosis have been important tools for elucidating the mechanisms of protection against cryptococcosis and for testing the efficacy of various antibody or antigen-based vaccine candidates against cryptococcal infection. Overall, animal models have provided a means for studying strategies for modulating T helper responses and evaluating antibodies and radioimmunotherapies. They have also proved useful in developing approaches for inducing cell responses using peptide mimotopes and specific cryptococcal antigens. Perhaps a shift in approach from defining targets for anti-fungal drug development toward devising immune therapies and/or vaccines that enhance host anti-fungal immune responses is a direction requiring more focus. Such studies will concentrate more on the host and less on the pathogen. Still, *Cryptococcus* is a disease in which the host and pathogen are intimately intertwined. Consequently, animal models will surely be required to understand the relationship and devise plausible treatments and/or vaccines to prevent cryptococcal infections.

## Conflict of Interest Statement

The authors declare that the research was conducted in the absence of any commercial or financial relationships that could be construed as a potential conflict of interest.

## References

[B1] Antachopoulos C. Walsh T. J. Roilides E. (2007). Fungal infections in primary immunodeficiencies. Eur. J. Pediatr. 166, 1099–1117 10.1007/s00431-007-0527-7 17551753

[B2] Arora S. Hernandez Y. Erb-Downward J. R. Mcdonald R. A. Toews G. B. Huffnagle G. B. (2005). Role of IFN-gamma in regulating T2 immunity and the development of alternatively activated macrophages during allergic bronchopulmonary mycosis. J. Immunol. 174, 6346–6356 15879135 10.4049/jimmunol.174.10.6346

[B3] Arora S. Olszewski M. A. Tsang T. M. McDonald R. A. Toews G. B. Huffnagle G. B. (2011). Effect of cytokine interplay on macrophage polarization during chronic pulmonary infection with *Cryptococcus neoformans*. Infect. Immun. 79, 1915–1926 10.1128/IAI.01270-10 21383052 PMC3088136

[B4] Beenhouwer D. O. Shapiro S. Feldmesser M. Casadevall A. Scharff M. D. (2001). Both Th1 and Th2 cytokines affect the ability of monoclonal antibodies to protect mice against *Cryptococcus neoformans*. Infect. Immun. 69, 6445–6455 10.1128/IAI.69.10.6445-6455.2001 11553589 PMC98780

[B5] Beenhouwer D. O. Yoo E. M. Lai C. W. Rocha M. A. Morrison S. L. (2007). Human immunoglobulin G2 (IgG2) and IgG4, but not IgG1 or IgG3, protect mice against *Cryptococcus neoformans* infection. Infect. Immun. 75, 1424–1435 10.1128/IAI.01161-06 17220317 PMC1828574

[B6] Biondo C. Messina L. Bombaci M. Mancuso G. Midiri A. Beninati C. Cusumano V. Gerace E. Papasergi S. Teti G. (2005). Characterization of two novel cryptococcal mannoproteins recognized by immune sera. Infect. Immun. 73, 7348–7355 10.1128/IAI.73.11.7348-7355.2005 16239533 PMC1273869

[B7] Bozzette S. A. Larsen R. A. Chiu J. Leal M. A. Jacobsen J. Rothman P. Robinson P. Gilbert G. Mccutchan J. A. Tilles J. (1991). A placebo-controlled trial of maintenance therapy with fluconazole after treatment of cryptococcal meningitis in the acquired immunodeficiency syndrome. California Collaborative Treatment Group. N. Engl. J. Med. 324, 580–584 10.1056/NEJM199102283240902 1992319

[B8] Brena S. Cabezas-Olcoz J. Moragues M. D. Fernandez De Larrinoa I. Dominguez A. Quindos G. Ponton J. (2011). Fungicidal monoclonal antibody C7 interferes with iron acquisition in *Candida albicans*. Antimicrob. Agents Chemother. 55, 3156–3163 10.1128/AAC.00892-10 21518848 PMC3122412

[B9] Bryan R. A. Jiang Z. Howell R. C. Morgenstern A. Bruchertseifer F. Casadevall A. Dadachova E. (2010). Radioimmunotherapy is more effective than antifungal treatment in experimental cryptococcal infection. J. Infect. Dis. 202, 633–637 10.1086/654813 20594103 PMC2904428

[B10] Casadevall A. (1995). Antibody immunity and invasive fungal infections. Infect. Immun. 63, 4211–4218 7591049 10.1128/iai.63.11.4211-4218.1995 PMC173598

[B11] Casadevall A. Cleare W. Feldmesser M. Glatman-Freedman A. Goldman D. L. Kozel T. R. Lendvai N. Mukherjee J. Pirofski L. A. Rivera J. Rosas A. L. Scharff M. D. Valadon P. Westin K. Zhong Z. (1998). Characterization of a murine monoclonal antibody to *Cryptococcus neoformans* polysaccharide that is a candidate for human therapeutic studies. Antimicrob. Agents Chemother. 42, 1437–1446 9624491 10.1128/aac.42.6.1437 PMC105619

[B12] Casadevall A. Mukherjee J. Devi S. J. Schneerson R. Robbins J. B. Scharff M. D. (1992). Antibodies elicited by a *Cryptococcus neoformans*-tetanus toxoid conjugate vaccine have the same specificity as those elicited in infection. J. Infect. Dis. 165, 1086–1093 10.1093/infdis/165.6.1086 1583327

[B13] Casadevall A. Pirofski L. A. (2007). Antibody-mediated protection through cross-reactivity introduces a fungal heresy into immunological dogma. Infect. Immun. 75, 5074–5078 10.1128/IAI.01001-07 17709417 PMC2168287

[B14] Centers for Disease Control and Prevention. (2010). Emergence of *Cryptococcus gattii* – Pacific Northwest, 2004–2010. Morb. Mortal. Wkly. Rep. 59, 865–868 10.1128/IAI.01001-07 20651641

[B15] Cheng P. Y. Sham A. Kronstad J. W. (2009). *Cryptococcus gattii* isolates from the British Columbia cryptococcosis outbreak induce less protective inflammation in a murine model of infection than *Cryptococcus neoformans*. Infect. Immun. 77, 4284–4294 10.1128/IAI.00176-09 19635827 PMC2747943

[B16] Cher D. J. Mosmann T. R. (1987). Two types of murine helper T cell clone. II. Delayed-type hypersensitivity is mediated by TH1 clones. J. Immunol. 138, 3688–3694 2953788

[B17] Cherwinski H. M. Schumacher J. H. Brown K. D. Mosmann T. R. (1987). Two types of mouse helper T cell clone. III. Further differences in lymphokine synthesis between Th1 and Th2 clones revealed by RNA hybridization, functionally monospecific bioassays, and monoclonal antibodies. J. Exp. Med. 166, 1229–1244 10.1084/jem.166.5.1229 2960769 PMC2189643

[B18] Chow S. K. Casadevall A. (2011). Evaluation of *Cryptococcus neoformans* galactoxylomannan-protein conjugate as vaccine candidate against murine cryptococcosis. Vaccine 29, 1891–1898 10.1016/j.vaccine.2010.12.134 21238568 PMC3043149

[B19] Clemons K. V. Brummer E. Stevens D. A. (1994). Cytokine treatment of central nervous system infection: efficacy of interleukin-12 alone and synergy with conventional antifungal therapy in experimental cryptococcosis. Antimicrob. Agents Chemother. 38, 460–464 10.1128/AAC.38.3.460 7911289 PMC284480

[B20] Cutler J. E. Deepe G. S. Jr. Klein B. S. (2007). Advances in combating fungal diseases: vaccines on the threshold. Nat. Rev. Microbiol. 5, 13–28 10.1038/nrmicro1537 17160002 PMC2214303

[B21] Dadachova E. Nakouzi A. Bryan R. A. Casadevall A. (2003). Ionizing radiation delivered by specific antibody is therapeutic against a fungal infection. Proc. Natl. Acad. Sci. U.S.A. 100, 10942–10947 10.1073/pnas.1731272100 12930899 PMC196907

[B22] Datta K. Bartlett K. H. Baer R. Byrnes E. Galanis E. Heitman J. Hoang L. Leslie M. J. Macdougall L. Magill S. S. Morshed M. G. Marr K. A. (2009a). Spread of *Cryptococcus gattii* into Pacific Northwest region of the United States. Emerging Infect. Dis. 15, 1185–1191 10.3201/eid1508.081384 19757550 PMC2815957

[B23] Datta K. Bartlett K. H. Marr K. A. (2009b). *Cryptococcus gattii*: Emergence in Western North America: Exploitation of a Novel Ecological Niche. Interdiscip. Perspect. Infect. Dis. 2009, 176532 10.1155/2009/176532 19266091 PMC2648661

[B24] Datta K. Lees A. Pirofski L. A. (2008). Therapeutic efficacy of a conjugate vaccine containing a peptide mimotope of cryptococcal capsular polysaccharide glucuronoxylomannan. Clin. Vaccine Immunol. 15, 1176–1187 10.1128/CVI.00130-08 18524882 PMC2519304

[B25] Devi S. J. (1996). Preclinical efficacy of a glucuronoxylomannan-tetanus toxoid conjugate vaccine of *Cryptococcus neoformans* in a murine model. Vaccine 14, 841–844 10.1016/0264-410X(95)00256-Z 8843625

[B26] Dong Z. M. Murphy J. W. (1995). Effects of the two varieties of *Cryptococcus neoformans* cells and culture filtrate antigens on neutrophil locomotion. Infect. Immun. 63, 2632–2644 7790079 10.1128/iai.63.7.2632-2644.1995 PMC173353

[B27] Dromer F. Mathoulin-Pelissier S. Fontanet A. Ronin O. Dupont B. Lortholary O. (2004). Epidemiology of HIV-associated cryptococcosis in France (1985-2001): comparison of the pre- and post-HAART eras. AIDS 18, 555–562 10.1097/00002030-200402200-00024 15090810

[B28] Fleuridor R. Lees A. Pirofski L. (2001). A cryptococcal capsular polysaccharide mimotope prolongs the survival of mice with *Cryptococcus neoformans* infection. J. Immunol. 166, 1087–1096 11145689 10.4049/jimmunol.166.2.1087

[B29] Galanis E. Macdougall L. (2010). Epidemiology of *Cryptococcus gattii*, British Columbia, Canada, 1999-2007. Emerging Infect. Dis. 16, 251–257 20113555 10.3201/eid1602.090900 PMC2958008

[B30] Gupta S. Ellis M. Cesario T. Ruhling M. Vayuvegula B. (1987). Disseminated cryptococcal infection in a patient with hypogammaglobulinemia and normal T cell functions. Am. J. Med. 82, 129–131 10.1016/0002-9343(87)90169-0 3492141

[B31] Hardison S. E. Wozniak K. L. Kolls J. K. Wormley F. L. Jr. (2010). Interleukin-17 is not required for classical macrophage activation in a pulmonary mouse model of *Cryptococcus neoformans* infection. Infect. Immun. 78, 5341–5351 10.1128/IAI.00845-10 20921149 PMC2981312

[B32] Huang C. Nong S. H. Mansour M. K. Specht C. A. Levitz S. M. (2002). Purification and characterization of a second immunoreactive mannoprotein from *Cryptococcus neoformans* that stimulates T-Cell responses. Infect. Immun. 70, 5485–5493 10.1128/IAI.70.10.5485-5493.2002 12228274 PMC128329

[B33] Huffnagle G. B. Lipscomb M. F. Lovchik J. A. Hoag K. A. Street N. E. (1994). The role of CD4^+^ and CD8^+^ T cells in the protective inflammatory response to a pulmonary cryptococcal infection. J. Leukoc. Biol. 55, 35–42 7904293 10.1002/jlb.55.1.35

[B34] Huffnagle G. B. Yates J. L. Lipscomb M. F. (1991). T cell-mediated immunity in the lung: a *Cryptococcus neoformans* pulmonary infection model using SCID and athymic nude mice. Infect. Immun. 59, 1423–1433 1825990 10.1128/iai.59.4.1423-1433.1991 PMC257859

[B35] Husain S. Wagener M. M. Singh N. (2001). *Cryptococcus neoformans* infection in organ transplant recipients: variables influencing clinical characteristics and outcome. Emerging Infect. Dis. 7, 375–381 10.3201/eid0701.010111 11384512 PMC2631789

[B36] Iseki M. Anzo M. Yamashita N. Matsuo N. (1994). Hyper-IgM immunodeficiency with disseminated cryptococcosis. Acta Paediatr. 83, 780–782 10.1111/j.1651-2227.1994.tb13140.x 7949815

[B37] Jain A. V. Zhang Y. Fields W. B. Mcnamara D. A. Choe M. Y. Chen G. H. Erb-Downward J. Osterholzer J. J. Toews G. B. Huffnagle G. B. Olszewski M. A. (2009). Th2 but not Th1 immune bias results in altered lung functions in a murine model of pulmonary *Cryptococcus neoformans* infection. Infect. Immun. 77, 5389–5399 10.1128/IAI.01079-08 19752036 PMC2786439

[B38] Jarvis J. N. Harrison T. S. (2008). Pulmonary cryptococcosis. Semin. Respir. Crit. Care Med. 29, 141–150 10.1055/s-2008-1063853 18365996

[B39] Kakeya H. Udono H. Ikuno N. Yamamoto Y. Mitsutake K. Miyazaki T. Tomono K. Koga H. Tashiro T. Nakayama E. Kohno S. (1997). A 77-kilodalton protein of *Cryptococcus neoformans*, a member of the heat shock protein 70 family, is a major antigen detected in the sera of mice with pulmonary cryptococcosis. Infect. Immun. 65, 1653–1658 9125543 10.1128/iai.65.5.1653-1658.1997 PMC175192

[B40] Kawakami K. Qifeng X. Tohyama M. Qureshi M. H. Saito A. (1996a). Contribution of tumour necrosis factor-alpha (TNF-alpha) in host defence mechanism against *Cryptococcus neoformans*. Clin. Exp. Immunol. 106, 468–474 10.1046/j.1365-2249.1996.d01-870.x 8973614 PMC2200622

[B41] Kawakami K. Tohyama M. Teruya K. Kudeken N. Xie Q. Saito A. (1996b). Contribution of interferon-gamma in protecting mice during pulmonary and disseminated infection with *Cryptococcus neoformans*. FEMS Immunol. Med. Microbiol. 13, 123–130 10.1111/j.1574-695X.1996.tb00225.x 8731020

[B42] Kawakami K. Tohyama M. Xie Q. Saito A. (1996c). IL-12 protects mice against pulmonary and disseminated infection caused by *Cryptococcus neoformans*. Clin. Exp. Immunol. 104, 208–214 10.1046/j.1365-2249.1996.14723.x 8625510 PMC2200435

[B43] Kleinschek M. A. Muller U. Schutze N. Sabat R. Straubinger R. K. Blumenschein W. M. Mcclanahan T. Kastelein R. A. Alber G. (2010). Administration of IL-23 engages innate and adaptive immune mechanisms during fungal infection. Int. Immunol. 22, 81–90 10.1093/intimm/dxp117 19951959

[B44] Kronstad J. W. Attarian R. Cadieux B. Choi J. D’souza C. A. Griffiths E. J. Geddes J. M. Hu G. Jung W. H. Kretschmer M. Saikia S. Wang J. (2011). Expanding fungal pathogenesis: *Cryptococcus* breaks out of the opportunistic box. Nat. Rev. Microbiol. 9, 193–203 10.1038/nrmicro2522 21326274 PMC4698337

[B45] Kwon-Chung K. J. Bennett J. E. (1984). Epidemiologic differences between the two varieties of *Cryptococcus neoformans*. Am. J. Epidemiol. 120, 123–130 6377880 10.1093/oxfordjournals.aje.a113861

[B46] Levitz S. M. Nong S. Mansour M. K. Huang C. Specht C. A. (2001). Molecular characterization of a mannoprotein with homology to chitin deacetylases that stimulates T cell responses to *Cryptococcus neoformans*. Proc. Natl. Acad. Sci. U.S.A. 98, 10422–10427 10.1073/pnas.181331398 11504924 PMC56976

[B47] Levitz S. M. Specht C. A. (2006). The molecular basis for the immunogenicity of *Cryptococcus neoformans* mannoproteins. FEMS Yeast Res. 6, 513–524 10.1111/j.1567-1364.2006.00071.x 16696647

[B48] Lutz J. E. Clemons K. V. Stevens D. A. (2000). Enhancement of antifungal chemotherapy by interferon-gamma in experimental systemic cryptococcosis. J. Antimicrob. Chemother. 46, 437–442 10.1093/jac/46.3.437 10980171

[B49] Maitta R. W. Datta K. Pirofski L. A. (2004). Efficacy of immune sera from human immunoglobulin transgenic mice immunized with a peptide mimotope of *Cryptococcus neoformans* glucuronoxylomannan. Vaccine 22, 4062–4068 10.1016/j.vaccine.2004.03.060 15364457

[B50] Mansour M. K. Schlesinger L. S. Levitz S. M. (2002). Optimal T cell responses to *Cryptococcus neoformans* mannoprotein are dependent on recognition of conjugated carbohydrates by mannose receptors. J. Immunol. 168, 2872–2879 11884457 10.4049/jimmunol.168.6.2872

[B51] Martinez L. R. Casadevall A. (2005). Specific antibody can prevent fungal biofilm formation and this effect correlates with protective efficacy. Infect. Immun. 73, 6350–6362 10.1128/IAI.73.10.6350-6362.2005 16177306 PMC1230912

[B52] May R. J. Beenhouwer D. O. Scharff M. D. (2003). Antibodies to keyhole limpet hemocyanin cross-react with an epitope on the polysaccharide capsule of *Cryptococcus neoformans* and other carbohydrates: implications for vaccine development. J. Immunol. 171, 4905–4912 14568972 10.4049/jimmunol.171.9.4905

[B53] Mcclelland E. E. Nicola A. M. Prados-Rosales R. Casadevall A. (2010). Ab binding alters gene expression in *Cryptococcus neoformans* and directly modulates fungal metabolism. J. Clin. Invest. 120, 1355–1361 10.1172/JCI38322 20335660 PMC2846037

[B54] Milam J. E. Herring-Palmer A. C. Pandrangi R. Mcdonald R. A. Huffnagle G. B. Toews G. B. (2007). Modulation of the pulmonary type 2 T-cell response to *Cryptococcus neoformans* by intratracheal delivery of a tumor necrosis factor alpha-expressing adenoviral vector. Infect. Immun. 75, 4951–4958 10.1128/IAI.00176-07 17646355 PMC2044519

[B55] Mitchell T. G. Perfect J. R. (1995). Cryptococcosis in the era of AIDS – 100 years after the discovery of *Cryptococcus neoformans*. Clin. Microbiol. Rev. 8, 515–548 8665468 10.1128/cmr.8.4.515 PMC172874

[B56] Mukherjee J. Nussbaum G. Scharff M. D. Casadevall A. (1995a). Protective and nonprotective monoclonal antibodies to *Cryptococcus neoformans* originating from one B cell. J. Exp. Med. 181, 405–409 10.1084/jem.181.1.405 7807020 PMC2191853

[B57] Mukherjee S. Lee S. C. Casadevall A. (1995b). Antibodies to *Cryptococcus neoformans* glucuronoxylomannan enhance antifungal activity of murine macrophages. Infect. Immun. 63, 573–579 7822024 10.1128/iai.63.2.573-579.1995 PMC173034

[B58] Muller U. Stenzel W. Kohler G. Werner C. Polte T. Hansen G. Schutze N. Straubinger R. K. Blessing M. Mckenzie A. N. Brombacher F. Alber G. (2007). IL-13 induces disease-promoting type 2 cytokines, alternatively activated macrophages and allergic inflammation during pulmonary infection of mice with *Cryptococcus neoformans*. J. Immunol. 179, 5367–5377 17911623 10.4049/jimmunol.179.8.5367

[B59] Murphy J. W. Mosley R. L. Cherniak R. Reyes G. H. Kozel T. R. Reiss E. (1988). Serological, electrophoretic, and biological properties of *Cryptococcus neoformans* antigens. Infect. Immun. 56, 424–431 3123390 10.1128/iai.56.2.424-431.1988 PMC259299

[B60] Nooney L. Matthews R. C. Burnie J. P. (2005). Evaluation of Mycograb, amphotericin B, caspofungin, and fluconazole in combination against *Cryptococcus neoformans* by checkerboard and time-kill methodologies. Diagn. Microbiol. Infect. Dis. 51, 19–29 10.1016/j.diagmicrobio.2004.08.013 15629225

[B61] Nussbaum G. Yuan R. Casadevall A. Scharff M. D. (1996). Immunoglobulin G3 blocking antibodies to the fungal pathogen *Cryptococcus neoformans*. J. Exp. Med. 183, 1905–1909 10.1084/jem.183.4.1905 8666947 PMC2192512

[B62] Olszewski M. A. Huffnagle G. B. Traynor T. R. Mcdonald R. A. Cook D. N. Toews G. B. (2001). Regulatory effects of macrophage inflammatory protein 1alpha/CCL3 on the development of immunity to *Cryptococcus neoformans* depend on expression of early inflammatory cytokines. Infect. Immun. 69, 6256–6263 10.1128/IAI.69.10.6256-6263.2001 11553568 PMC98759

[B63] Onishi R. M. Gaffen S. L. (2010). Interleukin-17 and its target genes: mechanisms of interleukin-17 function in disease. Immunology 129, 311–321 10.1111/j.1365-2567.2009.03240.x 20409152 PMC2826676

[B64] Pappas P. G. Alexander B. D. Andes D. R. Hadley S. Kauffman C. A. Freifeld A. Anaissie E. J. Brumble L. M. Herwaldt L. Ito J. Kontoyiannis D. P. Lyon G. M. Marr K. A. Morrison V. A. Park B. J. Patterson T. F. Perl T. M. Oster R. A. Schuster M. G. Walker R. Walsh T. J. Wannemuehler K. A. Chiller T. M. (2010). Invasive fungal infections among organ transplant recipients: results of the Transplant-Associated Infection Surveillance Network (TRANSNET). Clin. Infect. Dis. 50, 1101–1111 10.1086/649862 20218876

[B65] Park B. J. Wannemuehler K. A. Marston B. J. Govender N. Pappas P. G. Chiller T. M. (2009). Estimation of the current global burden of cryptococcal meningitis among persons living with HIV/AIDS. AIDS 23, 525–530 10.1097/QAD.0b013e328322ffac 19182676

[B66] Powderly W. G. (1993). Cryptococcal meningitis and AIDS. Clin. Infect. Dis. 17, 837–842 10.1093/clinids/17.5.837 8286622

[B67] Rachini A. Pietrella D. Lupo P. Torosantucci A. Chiani P. Bromuro C. Proietti C. Bistoni F. Cassone A. Vecchiarelli A. (2007). An anti-beta-glucan monoclonal antibody inhibits growth and capsule formation of *Cryptococcus neoformans* in vitro and exerts therapeutic, anticryptococcal activity in vivo. Infect. Immun. 75, 5085–5094 10.1128/IAI.00278-07 17606600 PMC2168274

[B68] Rivera J. Casadevall A. (2005). Mouse genetic background is a major determinant of isotype-related differences for antibody-mediated protective efficacy against *Cryptococcus neoformans*. J. Immunol. 174, 8017–8026 15944309 10.4049/jimmunol.174.12.8017

[B69] Rodrigues M. L. Shi L. Barreto-Bergter E. Nimrichter L. Farias S. E. Rodrigues E. G. Travassos L. R. Nosanchuk J. D. (2007). Monoclonal antibody to fungal glucosylceramide protects mice against lethal *Cryptococcus neoformans* infection. Clin. vaccine immunol. 14, 1372–1376 10.1128/CVI.00202-07 17715331 PMC2168121

[B70] Rosas A. L. Nosanchuk J. D. Casadevall A. (2001). Passive immunization with melanin-binding monoclonal antibodies prolongs survival of mice with lethal *Cryptococcus neoformans* infection. Infect. Immun. 69, 3410–3412 10.1128/IAI.69.5.3410-3412.2001 11292764 PMC98300

[B71] Saag M. S. Graybill R. J. Larsen R. A. Pappas P. G. Perfect J. R. Powderly W. G. Sobel J. D. Dismukes W. E. (2000). Practice guidelines for the management of cryptococcal disease. Infectious diseases society of America. Clin. Infect. Dis. 30, 710–718 10.1086/313757 10770733

[B72] Siddiqui A. A. Brouwer A. E. Wuthiekanun V. Jaffar S. Shattock R. Irving D. Sheldon J. Chierakul W. Peacock S. Day N. White N. J. Harrison T. S. (2005). IFN-gamma at the site of infection determines rate of clearance of infection in cryptococcal meningitis. J. Immunol. 174, 1746–1750 15661940 10.4049/jimmunol.174.3.1746

[B73] Singh N. Lortholary O. Alexander B. D. Gupta K. L. John G. T. Pursell K. Munoz P. Klintmalm G. B. Stosor V. Del Busto R. Limaye A. P. Somani J. Lyon M. Houston S. House A. A. Pruett T. L. Orloff S. Humar A. Dowdy L. Garcia-Diaz J. Kalil A. C. Fisher R. A. Husain S. (2005). An immune reconstitution syndrome-like illness associated with *Cryptococcus neoformans* infection in organ transplant recipients. Clin. Infect. Dis. 40, 1756–1761 10.1086/430606 15909263

[B74] Subramaniam K. S. Datta K. Quintero E. Manix C. Marks M. S. Pirofski L. A. (2010). The absence of serum IgM enhances the susceptibility of mice to pulmonary challenge with *Cryptococcus neoformans*. J. Immunol. 184, 5755–5767 10.4049/jimmunol.0901638 20404271 PMC2885446

[B75] Tabone M. D. Leverger G. Landman J. Aznar C. Boccon-Gibod L. Lasfargues G. (1994). Disseminated lymphonodular cryptococcosis in a child with X-linked hyper-IgM immunodeficiency. Pediatr. Infect. Dis. J. 13, 77–79 10.1097/00006454-199401000-00019 8170740

[B76] Taborda C. P. Casadevall A. (2001). Immunoglobulin M efficacy against *Cryptococcus neoformans*: mechanism, dose dependence, and prozone-like effects in passive protection experiments. J. Immunol. 166, 2100–2107 11160261 10.4049/jimmunol.166.3.2100

[B77] Taborda C. P. Rivera J. Zaragoza O. Casadevall A. (2003). More is not necessarily better: prozone-like effects in passive immunization with IgG. J. Immunol. 170, 3621–3630 12646626 10.4049/jimmunol.170.7.3621

[B78] Van Der Horst C. M. Saag M. S. Cloud G. A. Hamill R. J. Graybill J. R. Sobel J. D. Johnson P. C. Tuazon C. U. Kerkering T. Moskovitz B. L. Powderly W. G. Dismukes W. E. (1997). Treatment of cryptococcal meningitis associated with the acquired immunodeficiency syndrome. National Institute of Allergy and Infectious Diseases Mycoses Study Group and AIDS Clinical Trials Group. N. Engl. J. Med. 337, 15–21 10.1056/NEJM199707033370103 9203426

[B79] Vecchiarelli A. Pericolini E. Gabrielli E. Chow S. K. Bistoni F. Cenci E. Casadevall A. (2011). *Cryptococcus neoformans* galactoxylomannan is a potent negative immunomodulator, inspiring new approaches in anti-inflammatory immunotherapy. Immunotherapy 3, 997–1005 10.2217/imt.11.86 21843086

[B80] Vibhagool A. Sungkanuparph S. Mootsikapun P. Chetchotisakd P. Tansuphaswaswadikul S. Bowonwatanuwong C. Ingsathit A. (2003). Discontinuation of secondary prophylaxis for cryptococcal meningitis in human immunodeficiency virus-infected patients treated with highly active antiretroviral therapy: a prospective, multicenter, randomized study. Clin. Infect. Dis. 36, 1329–1331 10.1086/374849 12746781

[B81] Wormley F. L. Jr. Perfect J. R. Steele C. Cox G. M. (2007). Protection against cryptococcosis by using a murine gamma interferon-producing *Cryptococcus neoformans* strain. Infect. Immun. 75, 1453–1462 10.1128/IAI.00274-06 17210668 PMC1828544

[B82] Wozniak K. L. Hardison S. E. Kolls J. K. Wormley F. L. (2011a). Role of IL-17A on resolution of pulmonary *C*. *neoformans* infection. PLoS ONE 6, e17204 10.1371/journal.pone.0017204 21359196 PMC3040760

[B83] Wozniak K. L. Young M. L. Wormley F. L. (2011b). Protective immunity against experimental pulmonary *Cryptococcosis* in T cell-depleted mice. Clin. Vaccine Immunol. 18, 717–723 10.1128/CVI.00036-11 21450975 PMC3122518

[B84] Wozniak K. L. Ravi S. Macias S. Young M. L. Olszewski M. A. Steele C. Wormley F. L. (2009). Insights into the mechanisms of protective immunity against *Cryptococcus neoformans* infection using a mouse model of pulmonary cryptococcosis. PLoS ONE 4, e6854 10.1371/journal.pone.0006854 19727388 PMC2731172

[B85] Wright L. Bubb W. Davidson J. Santangelo R. Krockenberger M. Himmelreich U. Sorrell T. (2002). Metabolites released by *Cryptococcus neoformans* var. neoformans and var. gattii differentially affect human neutrophil function. Microbes Infect. 4, 1427–1438 10.1016/S1286-4579(02)00024-2 12475633

[B86] Yauch L. E. Lam J. S. Levitz S. M. (2006). Direct inhibition of T-cell responses by the *Cryptococcus* capsular polysaccharide glucuronoxylomannan. PLoS Pathog. 2, e120 10.1371/journal.ppat.0020120 17096589 PMC1635532

[B87] Yuan R. Casadevall A. Spira G. Scharff M. D. (1995). Isotype switching from IgG3 to IgG1 converts a nonprotective murine antibody to *Cryptococcus neoformans* into a protective antibody. J. Immunol. 154, 1810–1816 7836766

[B88] Yuan R. R. Casadevall A. Oh J. Scharff M. D. (1997). T cells cooperate with passive antibody to modify *Cryptococcus neoformans* infection in mice. Proc. Natl. Acad. Sci. U.S.A. 94, 2483–2488 10.1073/pnas.94.4.1437 9122221 PMC20114

[B89] Yuan R. R. Spira G. Oh J. Paizi M. Casadevall A. Scharff M. D. (1998). Isotype switching increases efficacy of antibody protection against *Cryptococcus neoformans* infection in mice. Infect. Immun. 66, 1057–1062 9488395 10.1128/iai.66.3.1057-1062.1998 PMC108015

[B90] Zaragoza O. Alvarez M. Telzak A. Rivera J. Casadevall A. (2007). The relative susceptibility of mouse strains to pulmonary *Cryptococcus neoformans* infection is associated with pleiotropic differences in the immune response. Infect. Immun. 75, 2729–2739 10.1128/IAI.00094-07 17371865 PMC1932903

[B91] Zaragoza O. Rodrigues M. L. De Jesus M. Frases S. Dadachova E. Casadevall A. (2009). The capsule of the fungal pathogen *Cryptococcus neoformans*. Adv. Appl. Microbiol. 68, 133–216 10.1016/S0065-2164(09)01204-0 19426855 PMC2739887

[B92] Zhang H. Zhong Z. Pirofski L. A. (1997). Peptide epitopes recognized by a human anti-cryptococcal glucuronoxylomannan antibody. Infect. Immun. 65, 1158–1164 9119446 10.1128/iai.65.4.1158-1164.1997 PMC175112

[B93] Zhang Y. Wang F. Tompkins K. C. Mcnamara A. Jain A. V. Moore B. B. Toews G. B. Huffnagle G. B. Olszewski M. A. (2009). Robust Th1 and Th17 immunity supports pulmonary clearance but cannot prevent systemic dissemination of highly virulent *Cryptococcus neoformans* H99. Am. J. Pathol. 175, 2489–2500 10.2353/ajpath.2009.090530 19893050 PMC2789623

[B94] Zhou Q. Gault R. A. Kozel T. R. Murphy W. J. (2006). Immunomodulation with CD40 stimulation and interleukin-2 protects mice from disseminated cryptococcosis. Infect. Immun. 74, 2161–2168 10.1128/IAI.74.3.1995.2006 16552046 PMC1418893

[B95] Zhou Q. Gault R. A. Kozel T. R. Murphy W. J. (2007). Protection from direct cerebral *cryptococcus* infection by interferon-gamma-dependent activation of microglial cells. J. Immunol. 178, 5753–5761 17442959 10.4049/jimmunol.178.9.5753

